# TUTORIAL-STYLE EDUCATION FOR UNDERGRADUATES IN AGING

**DOI:** 10.1093/geroni/igac059.1722

**Published:** 2022-12-20

**Authors:** Karley Deason, Nasreen Sadeq, Brianne Stanback

**Affiliations:** University of South Florida, Tampa, Florida, United States; University of South Florida, Tampa, Florida, United States; University of South Florida, Tampa, Florida, United States

## Abstract

At almost every university, there is an option for undergraduates to enroll in directed readings or research, independent studies, or some other form of tutorial-style education, although not much is known about them or best practices associated with them for undergraduate students interested in aging. This project reviews available literature about tutorial-style education and describes the multidimensions involved in these learning experiences. Search phrases such as “history of Oxford tutorials”, “tutorial classes”, and “student traits in tutorials” were used in Google Scholar to find literature, returning 142 articles. Articles were included if the literature was published in a periodical, findings were not redundant, and measures connected to the objectives of this project, and, by these criteria, ten articles comprised the final sample for review. Student characteristics, instructors, and other factors, like the time intensity of the tutorial course and the student’s connection to the subject area, have been found to impact tutorial-style education. The review is the first of its kind to use its findings to propose a set of best practices to enhance the experience for students interested in aging, including the fit of student and instructor, skillsets of successful students, and modalities, and advance a research agenda to better understand and communicate practices in tutorial-style education for undergraduates in aging.

